# Tipping Point Detection Using Reservoir Computing

**DOI:** 10.34133/research.0174

**Published:** 2023-07-03

**Authors:** Xin Li, Qunxi Zhu, Chengli Zhao, Xuzhe Qian, Xue Zhang, Xiaojun Duan, Wei Lin

**Affiliations:** ^1^ College of Science, National University of Defense Technology, Changsha, Hunan 410073, China.; ^2^ Research Institute of Intelligent Complex Systems and MOE Frontiers Center for Brain Science, Fudan University, Shanghai 200433, China.; ^3^ Shanghai Artificial Intelligence Laboratory, Shanghai 200232, China.; ^4^ School of Mathematical Sciences, SCMS, SCAM, and CCSB, Fudan University, Shanghai 200433, China.

## Abstract

Detection in high fidelity of tipping points, the emergence of which is often induced by invisible changes in internal structures or/and external interferences, is paramountly beneficial to understanding and predicting complex dynamical systems (CDSs). Detection approaches, which have been fruitfully developed from several perspectives (e.g., statistics, dynamics, and machine learning), have their own advantages but still encounter difficulties in the face of high-dimensional, fluctuating datasets. Here, using the reservoir computing (RC), a recently notable, resource-conserving machine learning method for reconstructing and predicting CDSs, we articulate a model-free framework to accomplish the detection only using the time series observationally recorded from the underlying unknown CDSs. Specifically, we encode the information of the CDS in consecutive time durations of finite length into the weights of the readout layer in an RC, and then we use the learned weights as the dynamical features and establish a mapping from these features to the system’s changes. Our designed framework can not only efficiently detect the changing positions of the system but also accurately predict the intensity change as the intensity information is available in the training data. We demonstrate the efficacy of our supervised framework using the dataset produced by representative physical, biological, and real-world systems, showing that our framework outperforms those traditional methods on the short-term data produced by the time-varying or/and noise-perturbed systems. We believe that our framework, on one hand, complements the major functions of the notable RC intelligent machine and, on the other hand, becomes one of the indispensable methods for deciphering complex systems.

## Introduction

Tipping point detection (TPD) has become one of the focal research topics in complex dynamical systems (CDSs) for decades, because it matches urgent needs arising in bioinformatics [1], climate [2], economics [3], and many other fields [4–6]. Mathematically, a tipping point is regarded as a critical state connecting the states before and after the bifurcation in a CDS [7]. However, the general concept of the tipping point is not only limited to the transition through a bifurcation point, but it could also be related to the abrupt transition caused by instantaneously internal switches of structures or/and alterations of internal parameters. In this context, because of the high complexity of dynamics and the observational noise of high dimension, detecting the transition points using traditional methods becomes fairly challenging. Here, we focus on the problem of TPD in the general sense for a given CDS.

A direct way to deal with this problem is to sensitively discover the change point of the temporal system based on the data of time series [8] or on the dynamic characteristics using some reconstruction techniques [9]. Actually, many detection methods and their theories on tipping points have been developed recently and systematically [10–12], which are roughly divided into 2 types: unsupervised and supervised methods. The unsupervised methods find tipping points usually through extracting the statistical features of the given data. A typical detection method of such a type is to analyze the probability distribution of the given data before and after the tipping point appears, and the logarithm of the likelihood ratio can be used for this detection [13]. To further improve the flexibility and broaden the applicability, the methods based on the density ratio estimation [14] and on the kernel functions [15,16] have been developed. In addition, the unsupervised detection methods also include the frameworks based on the Bayesian approach [17], the subspace [18], and the Markov chain [19]. Although these unsupervised methods perform well in some specific scenarios, they do not always sustain their accuracy in the face of the data collected from CDSs. The supervised methods require learning a mapping using the labeled dataset. In the previous works, some classifiers were used for this learning problem, including the support vector machine [20], the decision tree [21], the hidden Markov model [22], and the nearest neighborhood [23]. However, these supervised methods have not been developed extensively, because the labeled datasets of high quality are scarce and also because the tipping points usually emerge in highly unmeasurable and unpredictable manners in the time series produced by the unknown CDSs. Therefore, developing efficient methods is still extremely urgent, overcoming the difficulties encountered by the existing methods, to detect tipping points using the time series experimentally observed from the CDSs.

In this article, we utilize the reservoir computing (RC), a recently notable machine learning framework [24], to address the TPD problem in CDSs. Actually, the RC, which is a particular form of the recurrent neural network, was independently proposed in 2 contributions: the echo state networks [25] and the liquid state machines [26]. It is suitable for temporal data processing [27], while it requires less training data and faster learning speed than traditional methods in many tasks [28–30]. Naturally, the RC framework has good performances in pattern classification [31,32], time series forecasting [33,34], and system approximation [35]. In addition, recent studies suggest that the RC is able to classify the time series [36] and is also able to extract the dynamic parameters of the Lorenz system from the weights of its readout layer [37]. Inspired by all these advances, we are to identify the tipping points in a CDS through delicately measuring the fluctuations emergent in the RC weights.

Precisely, this article proposes a TPD framework using both the RC and the delicately designed statistical measures. The advantages of the proposed framework are summarized as follows.

1. We use the reservoir network to extract the stable features of the system and articulate a model-free and machine learning algorithm to detect changes, which requires less training data than the previous supervised methods demand but owns a better performance in detections than those standard methods have.

2. In the process of using machine learning algorithms to build the mapping from the features to system changes, we design a classification or a regression method. For the training set that contains the intensity information of system changes, the regression method can render not only the position of a tipping point detectable but also the intensity of the corresponding change estimable.

3. We integrate the sliding window into the detection process and make the detection method have the online capability. Indeed, we validate the generality and the flexibility of the proposed method in the CDSs containing noise with weak to moderate strengths and even in many real-world systems.

## Methods

The RC is a typical method suitable for processing temporal and spatial data. Previous works have demonstrated its ability in learning the inherent dynamics of a CDS [38]. When the internal structure of a considered system does not change, the RC can learn the dynamic characteristics using the data observed from the system. However, when the structures fluctuate with the time consistently or intermittently, the RC can also learn different dynamic characteristics sensitively along the time evolution. Using this advantage, we articulate a supervised framework, whose name is abbreviated as RC-TPD, to detect the tipping points in CDSs.

### Overall framework

**Fig. 1.**
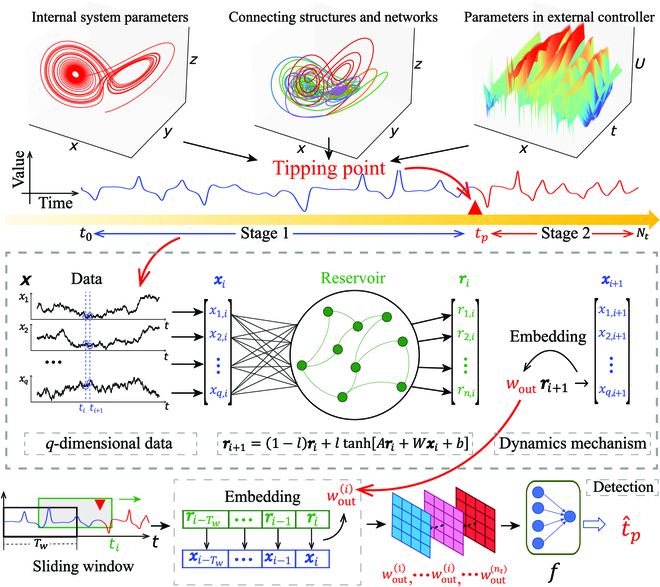
A sketch on applying the RC-TPD framework containing the RC and the sliding windows to locate the tipping point.

The framework of our model is sketchily depicted in Fig. 1. More specifically, a dynamical system is supposed to change its evolutionary dynamics from one typical characteristic to another at a certain time point *t_p_*, in that its essential parameters or structures are supposed to change at *t_p_*. Because of the complexity of the observed system, the presence of observational noise, and also the slow response of the dynamics induced by the structural change, it is often hard to identify the time point of the change directly and accurately from the data. Thus, we utilize the RC and the other appropriate machine learning methods to achieve this mission.

In general, the RC consists of 3 layers, i.e., the input layer, the hidden layer, and the output layer. The input *q*-dimensional temporal data ***X*** is embedded in a higher *n*-dimensional space to obtain the state sequence ***r****_t_*. Mathematically, for any time instant *t* = *i*, the dynamical evolution of the RC is represented by:

$$r_{i+1}=\left(1-l\right)\cdot r_{i}+l\cdot \text{tanh}\left(Ar_{i}+Wx_{i}+b\right),$$ (1)

where ***X*** = {***x***_1_, ***x***_2_, ⋯, ***x****_t_*, ⋯}, the reservoir network contains *n* nodes, *l* is the leakage parameter, *b* is the bias value, *W* is a matrix of *n* × *q* dimensions, *A* is the adjacency matrix for the reservoir network, and the matrices *W* and *A* are randomly generated and then fixed during the evolution of the system. Thus, the RC maps the reservoir state back to its *q*-dimensional data through a matrix *W*_out_ as:

$${\hat{x}}_{i+1}=W_{\text{out}}r_{i+1},$$ (2)

where $\hat{X}=\left\{{\hat{x}}_{1},{\hat{x}}_{2},\cdots ,{\hat{x}}_{t},\cdots \right\}$. A good RC machine can be trained to find the optimal *W*_out_ to minimize the difference between $\hat{X}$ and ***X***, that is, to minimize the loss function,

$$L=\sum_{i=0}^{T}‍{\left(W_{\text{out}}r_{i}-x_{i}\right)}^{2}+\beta ∥W_{\text{out}}∥,$$ (3)

where *T* is the data length, *β* > 0 is the regularization penalty term coefficient, and ∥ · ∥ represents an appropriate matrix norm, usually taking *L*_2_-norm. At this time, the RC machine encodes the dynamic information of the system to the weights *W*_out_ of the readout layer. When the system does not change, *W*_out_ can be regarded as a stable or unchanging feature of the system. After going through the tipping point, the system’s dynamics change, and *W*_out_ also changes correspondingly. Therefore, we are able to establish a function *f* mapping from *W*_out_ to the tipping point of the system by applying machine learning techniques. Here, the candidate models of machine learning can include the Logistic Regression, the Ridge Regression, the Support Vector Machine, the Random Forest, the Fully Connected Neural Network (FNN), and the Convolutional Neural Network (CNN).

### Algorithm details for 2 tasks

To achieve real-time detection of *W*_out_, a sliding window of length *T_w_* is introduced. As illustrated at the left lower panel of Fig. 1, at each time *i*, $W_{\text{out}}^{\left(i\right)}$ is obtained by applying the Ridge Regression [39] from the input data (***r****_i_*−*_T__w_*, …, ***r***_*i*−1_, ***r****_i_*) to the ouput data (***x***_*i*−*T_w_*_, …, ***x***_*i*−1_, ***x****_i_*). For a case where the system, although unknown, is uniformly invariant before the occurrence of a tipping point, it is sufficient to use the learned $W_{\text{out}}^{\left(i\right)}$ as a fixed feature to identify the tipping point.

However, for the most cases where the system’s dynamics before and after the tipping point are changing but the unknown system’s vector field is not deterministically prescribed, we need to quantify the variation of the adjacent *W*_out_ to identify the tipping point. Thus, for each time *i*, we define:

$$\Delta W_{\text{out}}^{\left(i\right)}=W_{\text{out}}^{\left(i+T_{w}\right)}-W_{\text{out}}^{\left(i\right)}.$$ (4)

Actually, depending on whether the change intensity information is contained in the training data, the mission could have 2 forms: the classification task and the regression task.

For the classification task, we generate *N* training data from the concerned system. For any time *t_i_* in each training data, set the feature as $\Delta W_{\text{out}}^{\left(i\right)}$ and flatten it to a vector. If the system does not change in the time period [*t*_*i*−*T_w_*_, *t*_*i*+*T_w_*_], then set the label as 0, and if the system changes at time *t_i_*, then set the label as 1. After training, we scan the test data with a sliding window of length 2*T_w_*. We input the obtained feature $\left(\Delta W_{\text{out}}^{\left(1\right)},\Delta W_{\text{out}}^{\left(i\right)},\ldots \right)$ into the classification model. When the system passes the tipping point, the probability of classification as 1 increases first and then decreases, and the abscissa of the maximum value is concluded as the predicted value, denoted by ${\hat{t}}_{p}$. In the experiments of the classification task, unless otherwise specified, we use the method of Random Forest [40] to approximate the detection function *f*. For the regression task, the essential idea is akin to that of the classification task. The only difference is that the changes of the intensity in the system can be quantified and used as the labels for the training data. Here, the changes are reflected by Δ*W*_out_. For this task, the training loss function is selected from the cross-entropy loss to the mean square error loss. In addition, when the sliding window passes through the tipping point in the test data, the output of our framework also produces a unimodal region. The abscissa of the peak is the predicted value ${\hat{t}}_{p}$ of the tipping point position, and the ordinate $\Delta \hat{h}$ is the intensity change value. In the experiments of the regression task, unless otherwise specified, we select the Ridge Regression method as the detection function *f* (see Appendix A for the pseudocode and a few additional instructions on the execution steps of the RC-TPD framework).

In what follows, we briefly explain the unimodal nature emergent in the output of our framework. Let ***X***(*t_i_*, *t_j_*) = (***x****_i_*, ***x***_*i*+1_, …, ***x****_j_*) and ***R***(*t_i_*, *t_j_*) = (***r****_i_*, ***r***_*i*+1_, …, ***r****_j_*). Then, $W_{\text{out}}^{\left(i\right)}$ is obtained by the linear regression from ***R***(*t*_*i*−*T_w_*_, *t_i_*) to ***X***(*t*_*i*−*T_w_*_, *t_i_*), denoted as $W_{\text{out}}^{\left(i\right)}=g\left(t_{i-T_{w}},t_{i}\right)$. We hypothetically set the system’s intensities before and after *t_p_* as *h*_1_ and *h*_2_ with *h*_1_ < *h*_2_ (for example, Δ*h* = *h*_2_ − *h*_1_ can be considered as a change in a system’s parameter). We first consider the situation to the left of the change time *t_p_*. For any *t*_*p*−*T_w_*_ < *t_j_* < *t_i_* < *t_p_*, combining the above notations with Eq. 4 yields:

$$\Delta W_{\text{out}}^{\left(i\right)}=g\left(t_{i},t_{i+T_{w}}\right)-g\left(t_{i-T_{w}},t_{i}\right)$$ (5)

and

$$\Delta W_{\text{out}}^{\left(j\right)}=g\left(\left(t_{j},t_{i}\right)\cup \left(t_{i},t_{j+T_{w}}\right)\right)-g\left(t_{j-T_{w}},t_{j}\right).$$ (6)

Because *W*_out_ is stable within the same dynamical system, the function *g* naturally exhibits translation invariance on the same side of the tipping point. Therefore, Eq. 5 can be rewritten as:

$$\Delta W_{\text{out}}^{\left(i\right)}=g\left(\left(t_{i},t_{j+T_{w}}\right)\cup \left(t_{j+T_{w}},t_{i+T_{w}}\right)\right)-g\left(t_{j-T_{w}},t_{j}\right).$$ (7)

Comparing Eqs. 6 and 7, we find that, from calculating *g*(*t_j_*, *t*_*j*+*T_w_*_) to calculating *g*(*t_i_*, *t*_*i*+*T_w_*_), the sample data with length (*i–j*) are converted to the system with intensity *h*_2_ after the tipping point. Therefore, using the trained *f*, we obtain that $f\left(\Delta W_{\text{out}}^{\left(i\right)}\right)-f\left(\Delta W_{\text{out}}^{\left(j\right)}\right)$ is greater than zero. This indicates that the detection output of the model monotonically increases in between [*t*_*p-T_w_*_, *t_p_*]. Analogously, it monotonously decreases in between [*t_p_*, *t*_*p*+*T_w_*_]. If *h*_1_ > *h*_2_, then the output distribution also exhibits a single peak characteristic, and the peak in this instance corresponds to a minimum point.

During the testing process, we slide the window by *δt* steps each time to detect the tipping point in real time. To enhance the efficiency of our framework, we can adopt a recursive least squares (RLS) strategy to train the reservoir computer and obtain $W_{\text{out}}^{\left(i\right)}$ at time *t_i_* [41]. Specifically, the RLS algorithm is given by:

$$\begin{array}{ll} & W_{\text{out}}^{\left(i\right)}=W_{\text{out}}^{\left(i-1\right)}+\frac{e_{i}r_{i}^{T}P_{i-1}}{1+r_{i}^{T}P_{i-1}r_{i}},\\ P_{i}=P_{i-1} & -\frac{P_{i-1}r_{i}r_{i}^{T}P_{i-1}}{1+r_{i}^{T}P_{i-1}r_{i}},e_{i}≔x_{i}-W_{\text{out}}^{\left(i-1\right)}r_{i}, \end{array}$$ (8)

where *P_i_* is a gain matrix, and its initial value is set to *P*_0_ = *I*/*γ*. Here, *I* ∈ *ℝ*^*N* × *N*^ represents the identity matrix, and *γ* > 0 is a constant parameter (we set *γ* = 0.01 in the following experiments).

## Results

### Demonstrations using synthetic data

For any dynamical system we investigated, we generate *N*-labeled data to train our model, and each data randomly selects the mutation point of the system parameters at time *t_p_* within the sampling length *N_t_*. To increase the difficulty of the detection, the initial values of the generated data and the changes in the system are randomly selected within a certain range. In addition, we also introduce the Gaussian random noise into the observational data, evaluating the robustness of the RC-TPD framework against noisy perturbations. First, we demonstrate the usefulness of the method by analyzing 3 representative physical systems whose dimensions are set from low to infinity.

To further evaluate the effectiveness and robustness of our model in detecting the tipping point, we compare it with several baseline methods under varying experimental conditions. Specifically, we compare our model with the following methods: (a) RC-TPD-R, a Ridge Regression method based on the dynamic features Δ*W*_out_; (b) RC-TPD-C, a Random Forest classification method based on the dynamic features Δ*W*_out_; (c) DATA-R, a Ridge Regression learning method based on the data features; (d) DATA-C, a Random Forest classification learning method based on data features; (e) FNN-R, a multilayer FNN method based on the data features for the regression task; (f) FNN-C, a multilayer FNN method based on the data features for the classification task; (g) CNN-R, a CNN method based on the data features for the regression task; (h) CNN-C, a CNN method based on the data features for the classification task; (i) KER, an unsupervised method based on the kernel [16]; and (j) DRE, an unsupervised method based on the density ratio estimation [13]. To guarantee a fair comparison, all of the aforementioned methods use the sliding window way for online detection. Methods (c) to (h) are supervised learning approaches that use time series data directly for training, while methods (i) and (j) are unsupervised learning methods that detect the tipping points based on the statistical characteristics of the data. For a clearer understanding of the above baseline methods (c) to (j), we include the implementation details in Appendix B.

#### The Lorenz63 system

The Lorenz system is a system proposed in 1963 to simulate the laws of atmospheric changes [42]. Therefore, timely detection of the tipping point where the system parameters change is of great significance to the prediction of system evolution. This representative system reads:

$$\dot{x}=\sigma \left(y-x\right),\dot{y}=\rho x-y-\mathrm{xz},\dot{z}=\mathrm{xy}-\zeta z,$$ (9)

where *x*, *y*, and *z* are 3 variables, and *σ*, *ρ*, and *ζ* are 3 parameters. We select the parameters as the values rendering system (h) chaotic, that is, *σ* = 10, *ρ* = 28, and *ζ* = 8/3.

We take the number of training sets as *N* = 500 and the number of test sets as 100. Each training data contains *N*_tr_ = 1,000 data points and each test data contains *N*_te_ = 5,000 data points with *dt* = 0.02. Moreover, the initial values of the 3 directions are all selected from a uniform distribution between 0 and 1. In addition, we take *T_w_* = 300 and randomly generate 2 numbers *σ*_1_, *σ*_2_ ∈ [5, 15]. Then, we change the system’s parameter *σ* from the number *σ*_1_ to *σ*_2_ at a randomly selected time *t_p_*, where *t_p_* ∈ [*T_w_*, *N_tr_* − *T_w_*] in training data and *t_p_* ∈ [2*T_w_*, *N*_te_ − 2*T_w_*] in test data. In fact, it is difficult to identify the tipping point of the system exclusively from the data itself or its simple transform (see Fig. S3 in Appendix D for an example). It, therefore, requires an efficient (parameter or network topology) sensitivity detection method, while the RC-TPD framework is a method of this kind.

**Fig. 2.**
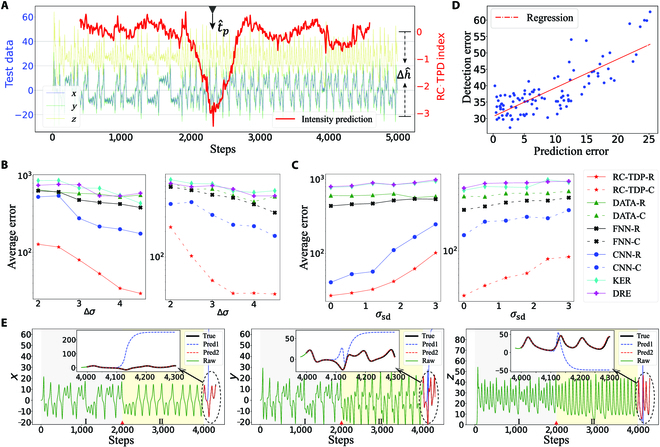
Experimental results of the Lorenz63 system (9). (A) Change of RC-TPD index with time in a test data, where Δ*σ* is −3.16. (B and C) The average detection errors obtained by using 10 different methods under different data conditions. (D) The relationship between the prediction effect and the detection effect in different reservoir settings. (E) Using the detected tipping point to assist the prediction of the next 300 steps using the RC, where the system parameter *σ* changes to 4 at *t_p_* = 2,000. Here, “Raw” corresponds to the original data, “True” stands for the real data for the next 300 steps, “Pred1” represents the result of using I∪II ([0, *N_t_*]) for prediction, and “Pred2” indicates the result only using II $\left([{\hat{t}}_{p},N_{t}]\right)$ for prediction.

The selection of hyperparameters during the training process is essential to unleashing the capability of the RC. We include the information on the detailed usage of the hyperparameters in Appendix C. The trained model is used to detect the tipping points in the test data. As shown in Fig. 2A, the RC-TPD framework can detect tipping points very sensitively, where the red line represents the RC-TPD index. For the regression task, our framework not only detects the position of the tipping point effectively but also predicts the intensity of parameter changes more accurately. The average error of position detection is 23.39 and the average error of intensity prediction is 0.304. For the classification task, even though the input information becomes less, the location of the tipping point also is detected, and the average error is 35.9. The experimental results presented above demonstrate the efficacy of our framework in detecting changes that are emergent in the underlying system. Additionally, when the system’s parameter changes across the bifurcation point of the parameter, the system’s dynamics often undergo significant changes. As such, it becomes necessary to detect the changes in the system as early and timely as possible. Our framework remains effective in this scenario (see Appendix D for experimental details and discussion).

To better evaluate the detection performance of our framework, we conduct a comparative analysis with a baseline method under varying experimental conditions. We consider a dataset consisting of 300 training sets and 100 test sets. First, for the test sets of different parameter changes Δ*σ* =  ∣ *σ*_2_ − *σ*_1_∣, our framework displays outstanding advantages and high resolving capability in detecting changes, as shown in Fig. 2B. Next, for different sizes of the observation noise standard deviation *σ*_sd_, our framework still exhibits accurate detection performance and strong robustness, as shown in Fig. 2C. In the situations where the number of training sets is limited, those methods based on the data characteristics may exhibit higher training errors. However, the RC-TPD framework can effectively learn the transformation law of the system even though available are the fewer training sets.

In fact, the detection effect of the RC method is closely related to its powerful predictive ability. Here we provide 2 experiments to explore their relationship. On one hand, when the reservoir learns more system dynamics characteristics, we believe that the reservoir can perform better in these 2 tasks. In other words, better predictions correspond to better detections, and vice versa. To verify this, we randomly generated the hyperparameters of the reservoir within a certain range and compared the relationship between the detection error and the prediction error of the reservoir. As shown in Fig. 2D, we randomly generated 100 reservoirs and gave the relationship between the detection errors and the prediction errors. Apparently, the detection error and the prediction error are positively correlated, so we can apply a suitable reservoir to solve these 2 tasks at the same time.

On the other hand, an efficient detection using the RC-TPD can also enhance the prediction accuracy and the prediction range. To illustrate this, we still use the Lorenz system (Eq. 8), where we generate the training data with *Nt* = 4,000 and *dt* = 0.01 and let the parameter *σ* change to another value at a certain moment. We use the RC-TPD framework to detect the tipping point *t_p_* and divide the time series into 2 parts (a) $\left[0,{\hat{t}}_{p}\right]$ and (b) $\left[\;{\hat{t}}_{p},\mathrm{Nt}\right]$, where ${\hat{t}}_{p}$ is the detected point. Then, we use the RC model to predict the next 300 steps of data. As shown in Fig. 2E, we use the whole data (that is, the data in the region I∪II without knowing the loci of the tipping point) as the input of the RC prediction model for prediction, and the prediction result is highlighted by “Pred1” and the prediction result obtained by only using the data in region II is indicated by “Pred2.” We change the system parameter *σ* at *t_p_* = 2,000, and no matter in which direction, the prediction performance only using the data in region II is obviously better. Therefore, as the data length is sufficient, tipping point identification implies a better predictive effect, although the data before and after *t_p_* have indistinguishable similarities to the naked eye.

#### The coupled Lorenz system

The coupled Lorenz system [43] is produced by coupling *m* individual oscillators of the Lorenz system, which reads

$$\begin{array}{ll} dx_{k}/\mathrm{dt} & =-10\left[x_{k}-y_{k}+c\sum_{l=1}^{m}‍a_{\mathrm{kl}}^{\left(x,y\right)}\left(y_{l}-y_{k}\right)\right],\\ dy_{k}/\mathrm{dt} & =28\left(1+h_{k}\right)x_{k}-y_{k}-x_{k}z_{k},\\ dz_{k}/\mathrm{dt} & =-\left(8/3\right)z_{k}+x_{k}y_{k}. \end{array}$$ (10)

Here, the coupling effect is contained in *a_kl_*. Among the coupled oscillators, the adjacency matrix *A* = {*a_ij_*} is a 0–1 matrix, and *c* is the coupling strength. The *h_k_* value makes the scales of these subsystems different.

**Fig. 3.**
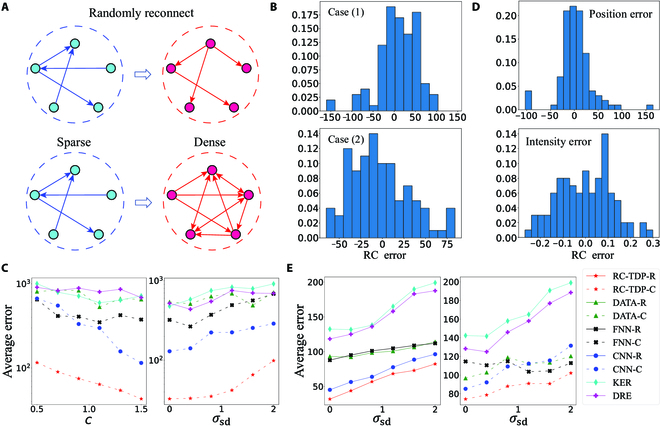
Experimental results of the coupled Lorenz system (Eq. 9) and the Kuramoto–Sivashinsky (KS) equation (10). (A) Two cases for changing the structure of the system are investigated: The edges of the subsystems are reconnected randomly and changed from sparse to the dense structure. (B) The obtained error distribution of the position detection in system (Eq. 9) for the cases in (A). (C) The average detection errors obtained by using 6 different methods under different data conditions in system (9). (D) The error distribution of the position detection and the intensity prediction of the tipping point obtained for the KS equation (Eq. 10). (E) The average detection errors obtained by using 10 different methods under different noise intensities in the KS equation 10.

Here, we consider the tipping point at which the coupling relationship between the subsystems changes. In particular, we set the adjacency matrix *A* having a change at *t_p_*. In addition, we take the number of training sets as *N* = 1,000 and the number of test sets as 100. Each training data contains *N*_tr_ = 1,000 data points and each test data contains *N*_te_ = 3,000 data points with *dt* = 0.002 and sampling time interval Δ*t* = 6*dt* = 0.012, and the initial value is randomly generated in the interval [−10, 10]. We randomly select a time instant to change the adjacency matrix *A*. Two types of changes are taken into account: (a) random reconnection and (b) changing matrix *A* from sparse to dense (see Fig. 3A). Because of the discontinuity induced by the structural change, we only use the classification task to solve this problem. We take *m* = 5, and the RC hyperparameters are listed in Table S3 of Appendix C. Then, we train the classifiers for each Lorenz system individually and use the predicted mean of the trained *m* classifiers as the detection signal. The prediction results are shown in Fig. 3B. Clearly, our framework can well distinguish the changes emergent in the system structure, and because the second case has a larger structure change, the detection performance seems to be even better. The average detection error for the first case is 38.89, while that for the second case is only 28.32.

To evaluate the effectiveness and the robustness of the RC-TPD framework, we compare 6 methods under the condition of random reconnection (excluding the 4 regression methods in the last example): (a) RC-TPD-C, (b) DATA-C, (c) FNN-C, (d) CNN-C, (e) KER, and (f) DRE. As shown in Fig. 3C, our framework outperformed the traditional methods for both cases, where *c* represents the coupling strength and *σ*_sd_ represents the standard deviation of the observational noise. Indeed, it is robust against the noise to a certain extent. In fact, the main reason for the poor performance of the other methods is that the Lorenz system is chaotic under the selected parameters, which certainly brings difficulties to the methods based only on the data features or statistical information.

#### Kuramoto–Sivashinsky equation

Finally, we consider the Kuramoto–Sivashinsky (KS) equation with spatiotemporally chaotic characteristics and add a spatial inhomogeneity term at the end of it [44], which reads:

$$u_{t}=-uu_{x}-u_{\mathrm{xx}}-u_{\text{xxxx}}+\mu \;\cos\left(\frac{2\pi x}{\lambda }\right).$$ (11)

Here, *μ* is the control parameter of the additional item, and *λ* is the wavelength. We randomly generate 2 control numbers *μ*_1_, *μ*_2_ ∈ [−1, 1] and consider the tipping point at which the system parameter *μ* transitions from *μ*_1_ to *μ*_2_ at a randomly selected time *t_p_*. In the simulation process, we integrate the KS equation (Eq. 10) on a grid of *Q* equally spaced points with *dt* = 0.01, so that we get *Q*-dimensional time series data. We take *λ* = 12, *Q* = 128, *N*_tr_ = 1,000, and *N*_te_ = 2,000 to generate 2,000 pieces of training data and 100 pieces of test data with sampling time interval Δ*t* = 20*dt* = 0.2. In addition, we take the observation error variance as 0.2. Moreover, we take *Q*^′^-dimensional data (*Q*^′^ = 32) at equal intervals as the features for training, and the results of the regression task are shown in Fig. 3D. Here, the average error of position detection is 22.66, and the average error of intensity prediction is 0.098. In the classification task, the average error of position detection is 34.84. Therefore, our framework also has a good performance for the KS equation, an infinitely dimensional system.

In addition, to demonstrate the robustness of our framework, we conduct experiments using the training dataset of a smaller size *N*_te_ = 500. We fix the parameter change Δ*μ* =  ∣ *μ*_1_ − *μ*_2_ ∣  = 0.5 in the test set for comparative analysis. Figure 3E shows the results of experiments conducted under different noise intensities. Because the changes before and after the tipping point are more prominent here, the 10 methods all exhibit certain detection abilities. Despite this, our framework, particularly the regression method, still exhibits the best detection performance. Additionally, when the noise is large, the supervised method based on data features has a detection ability similar to that of our RC-TPD-C. However, they are not as effective as the RC-TPD-R method. Thus, for the training data that contains intensity information, the regression task is expected to have better detection performance.

### Demonstrations using real-world data

The RC-TPD framework is so general and flexible that it can be applied to cope with many real-world problems. Although it is difficult to extract the internal operating mechanisms of the real systems from the data because of the strong randomness or/and the incomplete observations, our framework still successfully detects the tipping points in such systems. In what follows, we verify the efficacy of our framework using the datasets from 3 real-world experiments.

#### Character trajectories

First, we demonstrate the efficacy of our framework using the datasets of the character trajectories [45]. The dataset contains 2,858 handwritten character trajectories, and each trajectory is 3-dimensional, including 2-dimensional coordinate data and pen pressure data.

**Fig. 4.**
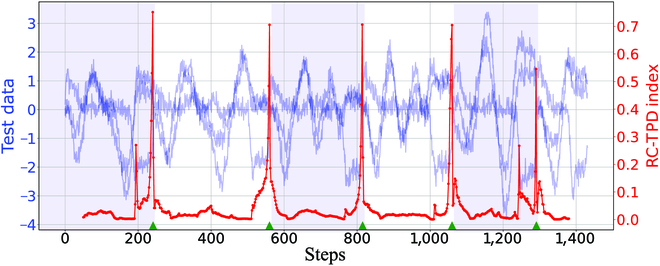
The detection effect of RC-TPD framework in character segmentation test data.

Before generating the training set, we first preprocess the data. We get rid of the parts that do not move at the beginning or the end of each data and then insert the mean between any 2 adjacent data points to expand the data. Finally, we splice any 2 character data into training data with segmentation point labels. Then, we splice several preprocessed character data into long test data. To increase the difficulty of segmentation detection, we introduce the Gaussian white noise with a mean value of 0 and a standard deviation of *σ*_sd_. We take the number of the training sets as 1,000, the number of the test sets as 100, the noise intensity as *σ*_sd_ = 0.2, and the window length as *T_w_* = 50. The detection effect of the RC-TPD framework is shown in Fig. 4. Clearly, this method can detect the position of the segmentation points (green triangle marks). We also test the other methods, and the average detection errors obtained by 6 different methods (RC-TPD, DATA, FNN, CNN, KER, and DRE) are 0.7, 1.5, 1.6, 2.2, 97.9, and 119.3, respectively.

According to the demonstration results, we find that, because the statistical distribution of the data before and after the split point does not change significantly, the performance of the unsupervised method is significantly worse than that of the supervised method. In fact, this detection task is not difficult for supervised methods. Thus, both the RC-TPD framework and the supervised methods based on data features can effectively detect the segmentation points. However, from the average of multiple demonstrations, the RC-TPD framework always attains a high detection accuracy and a stable detection performance. Hence, it is certain that our framework can be used in more complex real scenes.

#### Pediatric electroencephalogram (EEG) data

Patients with epilepsy suffer from recurrent seizures that occur at unpredictable times and usually without warning. Detecting epileptic seizures only using the EEG data can help us take active measures as early as possible and avoid more serious consequences for patients. The pediatric EEG data [46] used in the demonstration is available at http://physionet.org/physiobank/database/chbmit/.

**Fig. 5.**
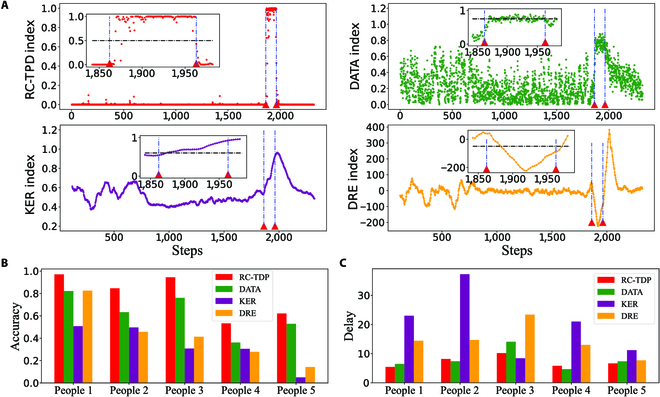
Detection performances using different methods in epileptic seizure data. (A) Changes in the detection indicators using 4 methods, including RC-TPD, DATA, KER, and DRE, in one test. (B) Comparison of detection accuracy using different methods in the data of 5 patients. (C) Comparison of response time delays using different methods in the data of 5 patients.

We regard the EEG data of the epilepsy without seizures as the benchmark system, so a sliding window with a length of *T_w_* can be used for detection. Here, we study the detection task for a specific patient, that is, train a detection model based on the patient’s limited epileptic seizure data, and the collected EEG signal contains 23 channels. We take the time window as 4 s (the sampling frequency is 256 Hz). To make a full use of the data, for each seizure cycle (*t*_start_, *t*_end_), we use a step size of *τ* to slide to obtain the positive training set as {(*t_i_*, *t_i_* + *T_w_*)| *t_i_* = *t*_start_ + *i* × *τ*, *t_i_* + *T_w_* ≤ *t*_end_}. Then, we randomly select the negative training set in the non-seizure area, the length and the number of which are selected in the same manner as those of the positive training set. Moreover, we take an extra second of data before training samples to warm up the RC machine. For each patient, we each time select one seizure data as the test set, and the rest of the data as the training set, that is, we use the cross-validation method to verify the detection effect. Similarly, we guarantee that the additional settings are fair, and we compare our framework with the other 3 methods, as shown in Fig. 5. More precisely, Fig. 5A reveals the fluctuations of the indicators over the time in a test sample of the 4 methods (see Appendix F for more details and results). Figure 5B and C presents the comparison of the average detection performances of the cross-validation in 5 patients, where the accuracy rate represents the ratio of the accurate warning time to the total warning time, and the response time represents the time from the onset of seizure to the warning.

From the demonstration results, we can see that, because of the strong randomness of the EEG data and the possibility of interference from other environmental factors, the detection accuracy of the unsupervised methods is not high. For the supervised methods, our framework can make better use of the limited training set than the DATA-C method to achieve the best detection accuracy and the lowest response time. In addition, our framework also has advantages compared to the other 2 methods based on the data features (CNN and FNN). Refer to Appendix F for details. Therefore, the supervised learning method we propose here can specifically detect the changes we care about in real-world data through training and can make full use of the training set to achieve more accurate and timely detection.

#### Tool wear detection data

Tool wear detection data comes from the 2010 PHM Society Conference Data Challenge [47]. The dataset contains 315 data files, and the data acquisition files are in .csv format, with 7 columns, corresponding to: (a) force in *X* dimension, (b) force in *Y* dimension, (c) force in *Z* dimension, (d) vibration in *X* dimension, (e) vibration in the *Y* dimension, (f) vibration in *Z* dimension, and (g) root mean square of acoustic emission. The spindle speed of the cutter was 10,400 RPM; the feed rate was 1,555 mm/min; the *Y* depth of cut (radial) was 0.125 mm; the *Z* depth of cut (axial) was 0.2 mm. Data were acquired at 50 kHz per channel. In addition, this dataset contains a “wear” file that lists wear after each cut in 10^−3^ mm. Therefore, different degrees of wear correspond to different sensor features. Below, we design a demonstration experiment to detect changes in the degree of wear.

**Fig. 6.**
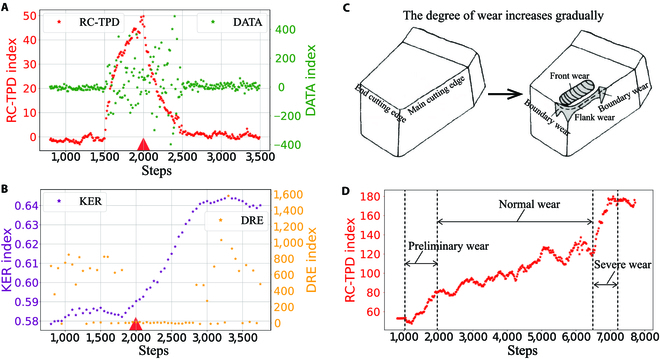
The detection performance using the RC-TPD framework in the tool wear detection data. (A and B) The performance diagram of 4 methods to detect the abrupt point of wear intensity. (C) Schematic diagram of tool wear. (D) Utilization of the RC-TPD framework to detect the wear of the tool under continuous working conditions.

Because the dataset contains intensity information (the degree of wear), we can use the regression task method RC-TPD-R to detect the tipping point. To generate training data, we created 1,000 data points with *N*_tr_ = 1300 and an observational noise of *σ*_sd_ = 1. Additionally, we generated 100 test data points with *N*_te_ = 4,000. Each piece of data contains a tipping point, and the data before and after this point correspond to different degrees of tool wear. We take *T_w_* = 500 and use the sliding window method to detect the test data, and the detection effect in a test experiment is shown in Fig. 6A and B. It is not difficult to see from the figure that the RC-TPD index can better estimate the position and the changing intensity of the tipping point based on the abscissa and the ordinate of the peak. Although the DATA indicator fluctuates when the sliding window passes the tipping point, it does not learn intensity information. Moreover, the other 2 unsupervised indicators cannot give appropriate judgments based on statistical characteristics. The detection error of the average position and the prediction error of the changing intensity using our framework in the test set are 77.6 and 5.48, respectively, which are significantly better than the results of 285 and 357 using the DATA method. In addition, our framework also has advantages compared to the other 2 methods based on the data features (CNN and FNN). Refer to Appendix G for more details and results.

In addition, the degree of tool wear gradually increases during use. As shown in Fig. 6C, tool wear owns 3 types, viz., front wear, flank wear, and boundary wear. According to the existing knowledge, the wear period can be divided into 3 stages: preliminary wear, normal wear, and severe wear. When the wear of the tool enters the severe wear stage, the wear of the tool is accelerated and the machining accuracy is greatly reduced, and the tool needs to be replaced. To detect the tipping point where the tool is scrapped, we use *W*_out_ with *T_w_* = 500 as the feature to predict the degree of tool wear. We generate 1,000 training sets and construct sensor data with gradually increasing wear and tear in accordance with experience as a test. As shown in Fig. 6D, our framework can predict the wear intensity very sensitively and clearly distinguish the 3 stages of the wear, so that the RC-TPD framework is able to detect the tipping point where the tool is about to be scrapped only through sensor data.

In fact, when the degree of tool wears changes, the data collected by each sensor operate in different operating mechanisms. Our framework can learn and detect the changes in this mechanism well, so it has an excellent detection ability.

## Discussion

The above experimental results clearly suggest that the RC-TPD framework proposed in this paper has an exceptional performance in tipping point detection tasks. Especially in deterministic systems, a good RC machine can encode the dynamics of the systems into the weights of the readout layer. Therefore, we feed the learned weights (Δ*W*_out_) as the dynamical features to machine learning algorithms to establish a function *f* connecting the features qualitatively or quantitively with system changes. Because these features under the same dynamics are approximately stable and invariant, it greatly reduces the overall space that needs to be learned. Therefore, it has higher resolving power and stronger robustness than unsupervised methods based on statistics, and it requires fewer training sets than traditional supervised methods based on time series data. In addition, when the target contains continuous intensity information, the regression method (RC-TPD-R) can accurately predict the location and the strength of the tipping point well at the same time. Otherwise, we use the classification method (RC-TPD-C) for location detection. Moreover, because the regression method has higher sensitivity to system changes, it brings more accurate position detection.

In addition, we can also find that the unsupervised method can only work when the test data changes significantly before and after the change point. However, our framework can use the learned dynamic characteristics for detection, so it has a stronger detection ability. This is mainly due to the powerful capability of the RC machines to process the time series data. Furthermore, we use the predictive performance of the RC machine to reflect the quality of learning system dynamics. From our demonstration experiments, we observe that better predictions correspond to improved detections, and better detections facilitate better predictions. Accordingly, it is reasonable to conclude that a good set of RC hyperparameters is applicable to different tasks based on the RC machine. However, this may serve as a limitation of our framework. We need to assume that the system has a stable underlying operating mechanism, and we must select appropriate RC hyperparameters to learn this mechanism. In fact, through experiments with real-world data, we find that this condition does not need to be satisfied in a rigorous sense, as long as it is approximately satisfied, our framework can show good performance in detection.

It is worth mentioning that the RC-TPD framework is completely based on data and can realize online detection by means of sliding windows. Therefore, the framework is general and has many points worthy of further in-depth study. First, it exhibits a high recognition rate and can be employed to detect both bifurcation points and parameter drift (see Appendices D and E). Therefore, it can be used to assist in detecting critical slowing down near the major changes. Second, because this framework can infer the instant where the structure connecting the subsystems changes, we can discover the temporal structure by using both the RC-TPD framework and the unidirectional or causal network reconstruction methods [48–50]. Finally, the framework has strong applicability, and it has a good detection effect even in real systems with strong randomness. We have conducted experiments on 3 real datasets. The experimental results show that this framework can embed the original data in the space of high dimension into the feature space that is easier for machine learning, so it also has good detection performance compared with those mainstream methods. Therefore, in the future work, this framework can be further combined with specific real scenarios and tasks to obtain more meaningful results. The abovementioned points are also focal issues of great concern at present.

In conclusion, by virtue of the advantages of RC, this work devotes to designing a model-free and machine learning framework for TPD. This method can efficiently detect the position of the tipping point by using the dynamic features and can predict the intensity of the change in the regression task. To verify the efficacy of the proposed method, we conducted experiments on the synthetic data from 3 simulation systems and on 3 real-world datasets as well. All the experimental results validate the higher detection performance of our framework over those traditional and representative methods. Indeed, it is shown that our framework requires less training data than the previous supervised methods. In addition, the algorithm framework is general and flexible, which, we believe, provides a theoretical basis for further and intensive study, and will be broadly applied to solving problems in real-world scenarios.

## Data Availability

The source codes and data will be made available on request. Moreover, the real datasets used in the manuscript could be accessed via https://archive-beta.ics.uci.edu/dataset/175/character+trajectories, http://physionet.org/physiobank/database/chbmit, and https://phmsociety.org/phm_competition/2010-phm-society-conference-data-challenge/.
