# Supplementary material for: Tipping Point Detection Using Reservoir Computing
Source: Research (Wash D C). 2023 Jul 3;6:0174. doi: 10.34133/research.0174 (PMC10317016; doi:10.34133/research.0174)
Supplement: Supplementary 1 — Appendix A to G Figs. S1 to S10 Tables S1 to S5 [file research.0174.f1.zip › RESEARCH-D-23-00176_SM.docx]

**Appendix**

**Appendix A:** **Implementation details of RC-TPD framework**

In Section II of the main text, we introduce the outline of the RC-TPD framework. Here, we include the execution steps and the related details of this method in combination with the corresponding pseudocode and sketch. As a machine learning method, its execution process is divided into two parts: training and testing, as shown in Algorithms 1, 2, and Fig. S1.

Here, Algorithm 1 presents the training part as implementing the RC-TPD framework, in which $reservoir.get\_states()$ means calculating intermediate states based on the input data, $\oplus$ represents the vector splicing symbol, and the function $model\_fit(X,Y)$ is employed to learn the mapping from *X* to *Y* using a suitable machine learning model. In our experiments, we utilized a ridge regression model for the regression task and a random forest model for the classification task. Algorithm 2 shows the testing part as implementing the RC-TPD framework, in which *len(X)* represents the length of the data *X*, *argmax(X)* represents the subscript of the largest element in *X*. In addition, in the process of the tipping point detection in the test set, in order to avoid the influence of the outliers, we select the peak top as the detection result according to the symmetrical single-peak characteristics of the model output. This thereby enhances the robustness of the method.

Accordingly, Figure S1 provides a clear visualization of the specific implementation details of our classification and regression algorithms. In this figure, we denote by *q* the input data dimension, $n+1$ as the parameter number of $\Delta W_{\mathrm{out}}$ (which includes 1 bias term), $T_{w}$ as the window length, $t_{p}$ as the position of the tipping point, and $\Delta h$ as the change intensity. Notably, when the above two tasks cross the tipping point, the RC-TPD indicator value tends to exhibit a single peak distribution, taking into account the output in the classification task, which represents the classification probability. At this point, the abscissa of the peak value represents the detection value of the tipping point, denoted as $\hat{t}_{p}$, whereas the abscissa of the peak value in the regression task indicates the predicted intensity change, represented by $\Delta\hat{h}$.

Fig. S1. Implementation details of the algorithm for detecting tipping points.

Table S1. Neural network architecture of FNN.

| 1 | nn.Linear(input_size, hidden_size, bias=True), nn.ReLU(), |
| --- | --- |
| 2 | nn.Linear(hidden_size, hidden_size, bias=True), nn.ReLU(), |
| 3 | nn.Linear(hidden_size, hidden_size, bias=True), nn.ReLU(), |
| 4 | nn.Linear(hidden_size, output_size, bias=True) |

In fact, according to different tasks, there are different options for the features extracted by the RC machine. In the above algorithm, we use $\Delta W_{\mathrm{out}}$ as the feature to detect the tipping point, which yields a good detection for deterministic systems. For those systems such as the benchmark dynamical systems, we even could directly use $W_{\mathrm{out}}$ as a feature in detection, which would not impair the detection accuracy. However, in many real systems with randomness, it is difficult for the RC to fully describe the internal operating mechanism of the investigated system. So, we suggest using $W_{1}\oplus W_{2}$ for feature extraction. Actually, no matter which feature is selected, the purpose of our framework is the same, that is, finding an accurate feature function mapping to the tipping point target using the machine learning technique. As for the specific application, we have provided a detailed explanation in the demonstration part of the main text.

## Appendix B: The baseline methods

To ensure a fair comparison, this article considers using online detection methods with a sliding window length of $T_{w}$ as the baseline methods. For the supervised methods, such as DATA, FNN, and CNN, the data within the sliding window is directly used as features for supervised learning. Specifically, the DATA-R method utilizes ridge regression to map the sliding window data to the system’s change intensity, while the DATA-C method uses random forests to classify the sliding window data into whether the system has undergone a change or not. The FNN method selects a FNN with three hidden layers, each layer has 64 hidden states, and the output dimension is 1 (see Tab. S1). The FNN-R method is used to predict the system’s change intensity, while the FNN-C method is used to predict whether the system has undergone a change. Similarly, the CNN method selects a neural network with two convolutional layers, one pooling layer, and two fully-connected layers, with an output dimension of 1 (see Tab. S2). The CNN-R method is used to predict the system’s change intensity, while the CNN-C method is used to predict whether the system has undergone a change. In fact, we also experiment with several similar structures for the FNN and CNN methods, such as increasing or decreasing the number of hidden layer neurons, and increasing or decreasing the number of layers in the network. However, it is observed that there is no significant difference in detection performance when the training data is determined.

The unsupervised methods KER and DRE employ the statistical concepts of kernel and density likelihood ratio to identify change points in the system. These methods exhibit strong performance in traditional change-point detection tasks. To achieve online detection, we execute the algorithm once for each input sliding window data. Despite their success in conventional settings, the statistical unsupervised methods may face limitations in the more complex scenario presented in this paper.

Table S2. Neural network architecture of CNN.

| 1 | nn.Conv1d(input_channels,16, 3, 1, 1), nn.ReLU(), |
| --- | --- |
| 2 | nn.MaxPool1d(2, stride=3), |
| 3 | nn.Conv1d(16, 4, 3, 1, 1), nn.ReLU(), |
| 4 | nn.Flatten(), |
| 5 | nn.Linear($\sim$, 32), nn.ReLU(), nn.Linear(32, output_dim) |

Table 3. Hyperparameters for the RC-TPD framework.

|  | *N* | *n* | *r* | *s* | *l* | *c* | $\sigma_{b}$ | $\alpha$ |
| --- | --- | --- | --- | --- | --- | --- | --- | --- |
| Lorenz63 (R) | 300 | 200 | 0.8 | 0.1 | 0.6 | 0.05 | 1.5 | 5 |
| Lorenz63 (C) | 600 | 200 | 0.8 | 0.1 | 0.6 | 0.05 | 1.5 | 5 |
| CL63 (C) | 1000 | 500 | 0.8 | 0.05 | 0.6 | 0.05 | 1 | 1 |
| KS (R) | 500 | 500 | 0.8 | 0.3 | 0.8 | 0.02 | 1.5 | 5 |
| KS (C) | 1000 | 500 | 0.8 | 0.3 | 0.1 | 0.02 | 1 | 5 |
| Real data1 | 1000 | 500 | 0.6 | 0.1 | 0.6 | 0.1 | 1 | 10 |
| Real data2 | Not unique | 500 | 0.8 | 0.2 | 0.6 | 0.05 | 0.5 | 5 |
| Real data3 | 1000 | 200 | 0.8 | 0.01 | 0.6 | 0.05 | 0.5 | 10 |

## Appendix C: Hyperparameter settings for different experiments

The prediction performance of reservoir computing is significantly influenced by its hyperparameters. Hence, it is imperative to select appropriate parameters for different systems, which include the number of nodes in the reservoir network (*n*), the spectral radius (*r*), the input scaling (*s*), the leaky rate (*l*), the connectivity (*c*), the bias term ($\sigma_{b}$), and the ridge regression regularization coefficient ($\alpha$). Additionally, the length of the sliding window ($T_{w}$) is also a crucial hyperparameter. In order to clearly present the selected RC hyperparameters in this experiment, we have provided a detailed list of them in Tab. S3.

Here, we examine the selection of hyperparameters, using the important hyperparameter $T_{w}$ as an example. A longer window length yields a more precise representation of the dynamic information contained in $W_{\mathrm{out}}$, thereby enhancing detection performance. However, for online detection tasks that depend on data from a time window after $t_{p}$, a longer window length may negatively impact timeliness. Conversely, if the window length is too short, it may not fully capture dynamic information, leading to difficulty in learning detection tasks. Therefore, selecting an appropriate window length is critical for our framework ology. It is worth noting that when the window data is adequate for RC to learn dynamic characteristics, increasing the window length does not significantly improve detection accuracy. As such, selecting the shortest time window length within an allowable detection error range suffices.

Fig. S2. Diagram showing the variation of position detection error of the tipping point and intensity prediction error of the system change with respect to $T_{w}$.

To validate the analysis presented above, we perform experiments on the Lorenz63 system with varying window lengths. The objective of these experiments is to investigate the correlation between window length and the accuracy of detecting $t_{p}$, as well as predicting $\Delta h$. The experimental outcomes, depicted in Fig. S2, are in agreement with our analysis, hence we select $T_{w}=300$ for this experiment.

## Appendix D: Detecting system changes through bifurcation points

Prior to conducting the experiment, it is imperative to discuss the differences and the connections between our defined generalized tipping-point detection and traditional bifurcation point detection. As stated in the introductory paragraph, we consider a more comprehensive scenario. The aim of this study is to detect a transition point in the underlying operating mechanism of a system, which can be triggered by internal changes or external interference, including but not limited to, transitions through bifurcation points. Therefore, based on specific learning tasks, we define tipping points as changes in the underlying system that we are concerned with. However, identifying the transition points efficiently using traditional methods can be challenging due to the high complexity of dynamics and the presence of high observational noise. For example, in the case of a specific change in the system parameter $\sigma$ of the chaotic Lorenz system at time $t_{p}$, we recorded observation data and generated their first and second-order difference diagrams. As depicted in Fig. S3, it is difficult to identify the precise location of $t_{p}$ through naked-eye inspection or traditional analysis methods. Therefore, our proposed dynamic-based detection method is of significant importance.

In reality, when a system reaches a bifurcation point, it undergoes an irreversible transition to a completely distinct state. An example of this can be seen in the Lorenz63 system, as illustrated in the left panel of Fig. S4, where the bifurcation occurs at a parameter value of approximately 24.74. While some conventional and straightforward methods can effectively identify this situation, there are instances, as depicted in the right panel of Fig. S4, where the bifurcation point may not be immediately apparent due to a time lag after the system parameters surpass the bifurcation point. Therefore, it remains essential to accurately detect the bifurcation point in a timely manner.

Fig. S3. Adding a tipping point to the Lorenz63 system. (a) The Lorenz63 system is sensitive to the small changes. (b) After introducing the tipping point and random observation noise. (c) The test data changes over time in three directions. (d) The first-order difference of the test data changes over time. (e) The second-order difference of the test data changes over time. The red triangle in (b)-(e) is the position of the tipping point and the parameter $\sigma$ changes from 8 to 11.48.

Fig. S4. The bifurcation behavior of the Lorenz63 system with parameter $\rho$. When the value of parameter $\rho$ is smaller than approximately 24.74, the system undergoes a transition from having three fixed points (chaos) to having a single fixed point. Depending on the specific settings and conditions, the system may exhibit a rapid transition to a completely different dynamics (left) or a slow transition (right).

Fig. S5. (a) The system immediately converges to a fixed point after the bifurcation point. (b) The system converges to a fixed point after a long delay following the bifurcation point.

Hence, in the context of a system undergoing transition at the bifurcation point, utilizing the classification method with $W_{\mathrm{out}}^{(i)}$ as the feature proves to be a valuable approach. Our proposed RC-TPD-C framework adeptly maps the dynamic feature to the parameter space and effectively learns appropriate thresholds to accurately classify the two distinct states that emerge post-bifurcation. To this end, we assign the state label of 0 before the bifurcation point and 1 after the bifurcation point. We generate 1000 training sets and 100 test sets to evaluate the efficacy of our framework. The experimental results, as depicted in Fig. S5, demonstrate that our framework exhibits timely detection performance even in bifurcation transition systems with long delays, with an average detection error of only 36.2.

## Appendix E: Detection of parameter drift

In many practical situations, the parameters of the system do not change suddenly at a certain point, but drift slowly over time. In these situations, it becomes more difficult to detect the changes along with time series data. In the main text, we have verified that the RC-TPD framework can attain a high detection fidelity. Here, we are to verify the detection effect of the method under the condition of parameter drift.

Fig. S6. The detection performances using the four methods (RC-TPD, DATA, KER, and DRE) to the Lorenz system (8) with the drifting parameter $\sigma$ specified in (E1). Here, the green triangles represent the start and the end times of the parameter drift, the blue line represents the changes in the three components of the system, and the red line represents the detection index.

First, we demonstrate our framework still using the Lorenz system (8), and the training set settings are the same as those in the main text. In the test set, we randomly select two points $t_{p1}$ and $t_{p2}$, and set the parameter $\sigma$ in system (8) changes as follows:

$\sigma(t)=\left\{ \begin{matrix} \sigma_{1}, & 0\leq t<t_{p1}, \\ \frac{1}{t_{p2}-t_{p1}}(\sigma_{2}-\sigma_{1})(t-t_{p1})+\sigma_{1}, & t_{p1}\leq t<t_{p2}, \\ \sigma_{2}, & t_{p1}\leq t<N_{te}. \end{matrix} \right.$ (E1)

We apply the four methods to the test set, respectively, and compare their results, as shown in Fig. S6. Due to the small change of the parameter $\sigma$ within the time window, the data-based, supervised method cannot identify the change of the system. However, the two unsupervised methods can detect this change over a long time span, but they cannot locate the start and end positions of the parameter drift. This shows that our framework has a strong detection ability in this deterministic system.

In order to further strengthen the above demonstration, we carry out similar experiments for the KS equation (10). The parameter $\mu$ in the test set is set as follows:

$\mu(t)=\left\{ \begin{matrix} \mu_{1}, & 0\leq t<t_{p1}, \\ \frac{1}{t_{p2}-t_{p1}}(\mu_{2}-\mu_{1})(t-t_{p1})+\mu_{1}, & t_{p1}\leq t<t_{p2}, \\ \mu_{2}, & t_{p1}\leq t<N_{\mathrm{te}}. \end{matrix} \right.$ (E2)

The experimental results are shown in Fig. S7. Clearly, the performance of using the RC-TPD framework attains the best level in the detection task of the parameter drift. Consequently, the above two examples together demonstrate the efficacy of our framework in realistic situations.

Fig. 7. Detection performances using four different methods (viz., RC-TPD, DATA, KER, DRE) in the KS equation (10) with the drifting parameter $\mu$ specified in (E2). Here, the symbols have the same meanings as those in Fig. S6.

## Appendix F: Supplementary instructions for seizure detection

Detecting epileptic seizures based on the EEG data is of clinical importance. Here we select 5 individuals to train the specific detection machines to evaluate our framework. For the supervised method, we have already given the way to generate the training set in the main text. However, in the training process, adopting different training models leads to different learning performances. Here, the RC-TPD framework directly uses the logistic regression to achieve the detections, but in the DATA method, only the random forest method can be used. For the unsupervised methods, due to the timeliness of the epileptic seizure detection, we choose a suitable sliding window so that both the KER and the DRE methods can achieve online detection. In short, these settings ensure the fairness and the reliability of the comparison study for different methods.

We take patient 1 as an example to introduce the results of the cross-validation experiment. We present, in Fig. S8, the detection indicators using the four methods (RC-TPD, DATA, KER, and DRE) for the cross-validation. In addition, we set appropriate alarm thresholds based on the detection accuracy and the response time, and finally, evaluate these methods based on the calculated average detection accuracy and the average response time. Clearly, in addition to the epileptic seizures, EEG data may also fluctuate due to the other conditions. our framework can eliminate some other fluctuations, so as to specifically detect the fluctuations caused by seizures. When we use the DRE method, selecting appropriate kernel parameters is beneficial to identifying the seizures. However, this method requires a longer window length, so in order to ensure the response time, the response threshold is often small and then the detection accuracy is not that high. Therefore, all these suggest that our framework can extract changing features in some random dynamical system, which is conducive for training the detection model.

Fig. S8. Test indicators for cross-validation using different methods.

Moreover, despite the utilization of advanced neural network models in FNN and CNN, which are based on data features, they are unable to attain comparable outcomes as our proposed method due to the restricted availability of data, as illustrated in Fig. 15. Consequently, it is evident that dynamic feature-based detection is a viable solution even in intricate task scenarios characterized by the noise of high levels.

## Appendix G: Supplementary instructions for tool wear detection

First, we preprocess the data for tool wear detection. To do so, we remove the unstable data during the initial insertion of the tool. Then collect a data point every three points to compress the data. Finally, the data of different degrees of wear are spliced together as the training and the test sets. In the main text, we generate 1,000 training data and 100 test data for the first tool wear data. The error distributions of the position detection and the intensity prediction are shown in Fig. S10, where $t_{p}\in[800,3700]$ and $\Delta h\in[39.64,125.53]$. Clearly, the errors of the position detection and the intensity prediction approximately obey a normal distribution with zero mean. This indicates that the results obtained by using our framework attain the lowest error compared to the results using the other methods.

Fig. S9. Comparison of detection accuracy and response time delays using different methods, including FNN and CNN, in the data of five patients.

Fig. S10. The error distribution diagrams of position detection and strength prediction in the test data of tool wear data.

To enhance the credibility of the RC-TPD framework in this dataset, we conduct additional tests on the second and the third tool wear data. Furthermore, we incorporate the FNN and the CNN techniques to the baseline methods to augment our analysis. The results are shown in Tabs. S4-S5, showing that our framework has the best performance. In fact, tools with different degrees of wear show different vibration modes and force distributions when working. This difference can be sensitively captured by our framework, so that the tipping point can be accurately detected. Therefore, our framework can exert powerful capabilities in addressing problems for many real-world systems.

Table S4. Position detection performances of three tool data using different methods.

| Method | Tool 1 | Tool 2 | Tool 3 |
| --- | --- | --- | --- |
| RC-TPD | 77.6 | 71.2 | 62.4 |
| DATA | 285 | 238 | 294 |
| KER | 603 | 567 | 621 |
| DRE | 832 | 736 | 764 |
| FNN | 484 | 258 | 504 |
| CNN | 232 | 274 | 484 |

Table S5. Intensity prediction performances of three tool data using different methods.

| Method | Tool 1 | Tool 2 | Tool 3 |
| --- | --- | --- | --- |
| RC-TPD | 4.09 | 5.06 | 6.74 |
| DATA | $>$100 | $>$100 | $>$100 |
| KER | invalid | invalid | invalid |
| DRE | invalid | invalid | invalid |
| FNN | 41 | 18 | 37 |
| CNN | 12 | 11 | 31.2 |
