# Supplementary material for: Tipping Point Detection Using Reservoir Computing
Source: Research (Wash D C). 2023 Jul 3;6:0174. doi: 10.34133/research.0174 (PMC10317016; doi:10.34133/research.0174)
Supplement: Supplementary 1 — Appendix A to G Figs. S1 to S10 Tables S1 to S5 [file research.0174.f1.zip › Fig-S1.pdf]

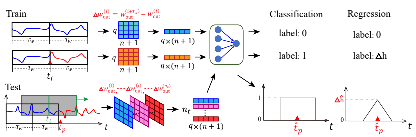

Train
$\Delta w_{\text{out}}^{(i)} = w_{\text{out}}^{(i+T_w)} - w_{\text{out}}^{(i)}$
Classification
Regression
T_w
T_w
q
$n+1$
$q \times (n+1)$
label: 0
label: 0
t_i
label: 1
label: Δh
Test
$\Delta w_{\text{out}}^{(i)}, \cdots \Delta w_{\text{out}}^{(i)}, \cdots \Delta w_{\text{out}}^{(n_t)}$
n_t
t_i
t_p
t
T_w
$q \times (n+1)$
1
0
$\hat{t}_p$
t
$\Delta\hat{h}$
0
$\hat{t}_p$
t
