# Supplementary figures and images for: Tipping Point Detection Using Reservoir Computing
Source: Research (Wash D C). 2023 Jul 3;6:0174. doi: 10.34133/research.0174 (PMC10317016; doi:10.34133/research.0174)

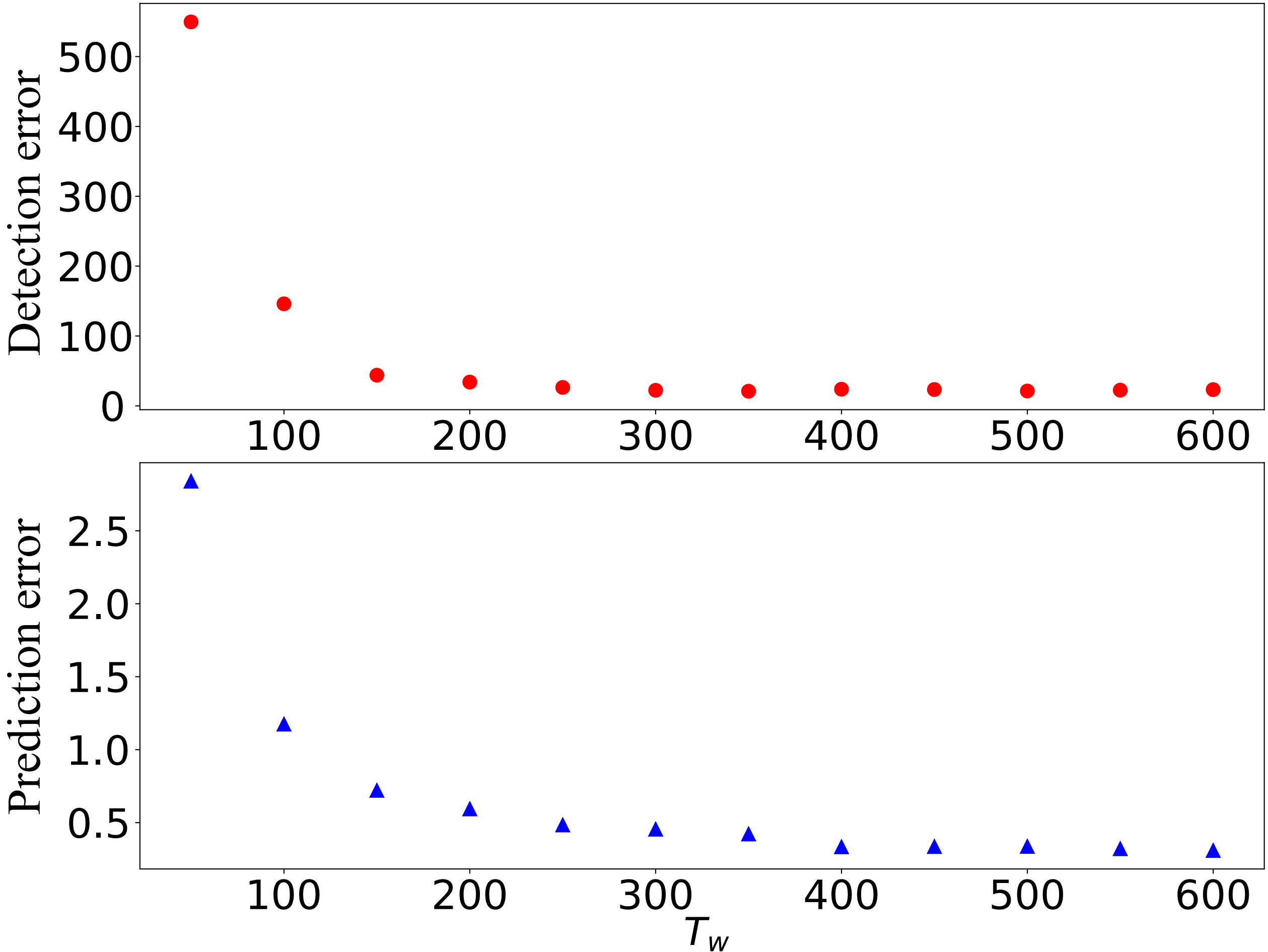

Supplement: Supplementary 1 — Appendix A to G Figs. S1 to S10 Tables S1 to S5 [file research.0174.f1.zip › Fig-S2.pdf]

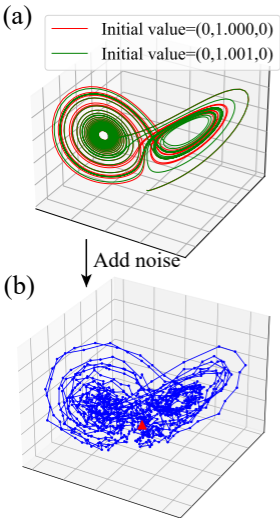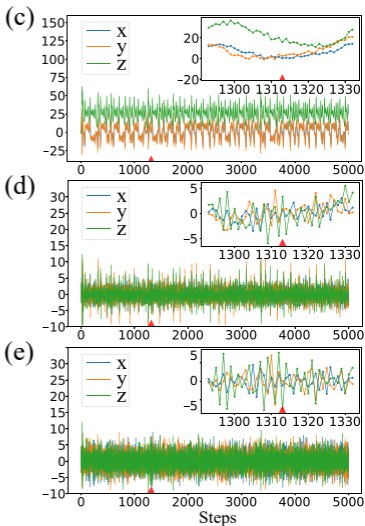

Supplement: Supplementary 1 — Appendix A to G Figs. S1 to S10 Tables S1 to S5 [file research.0174.f1.zip › Fig-S3.pdf]

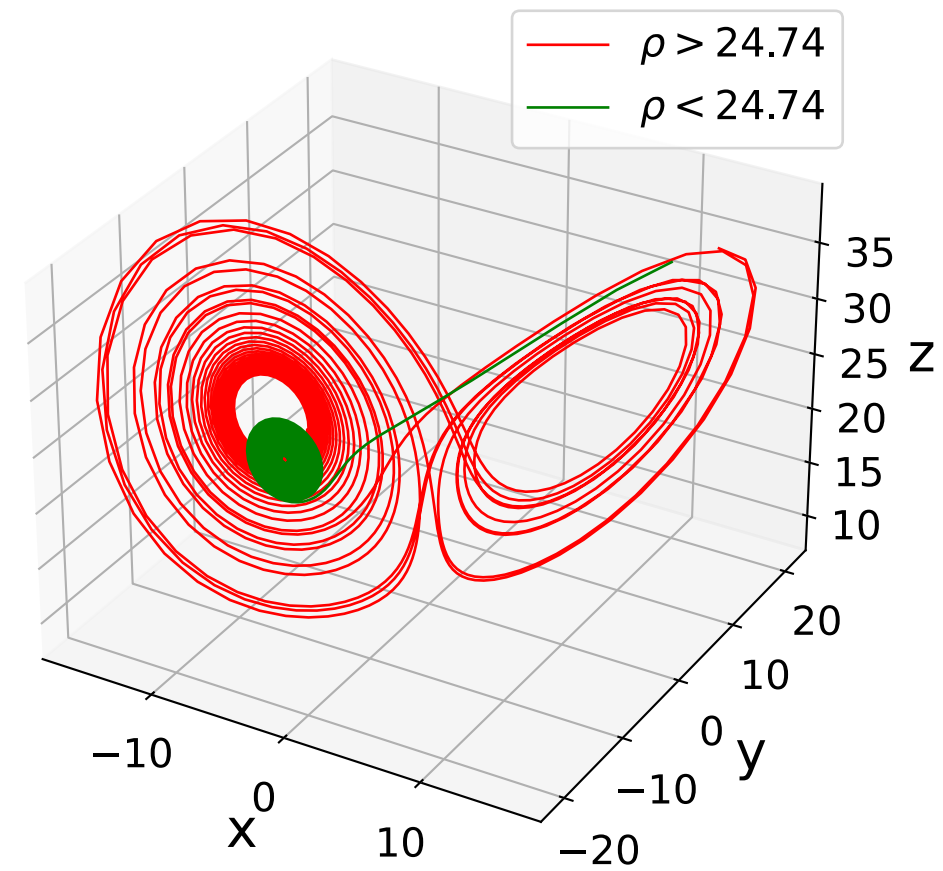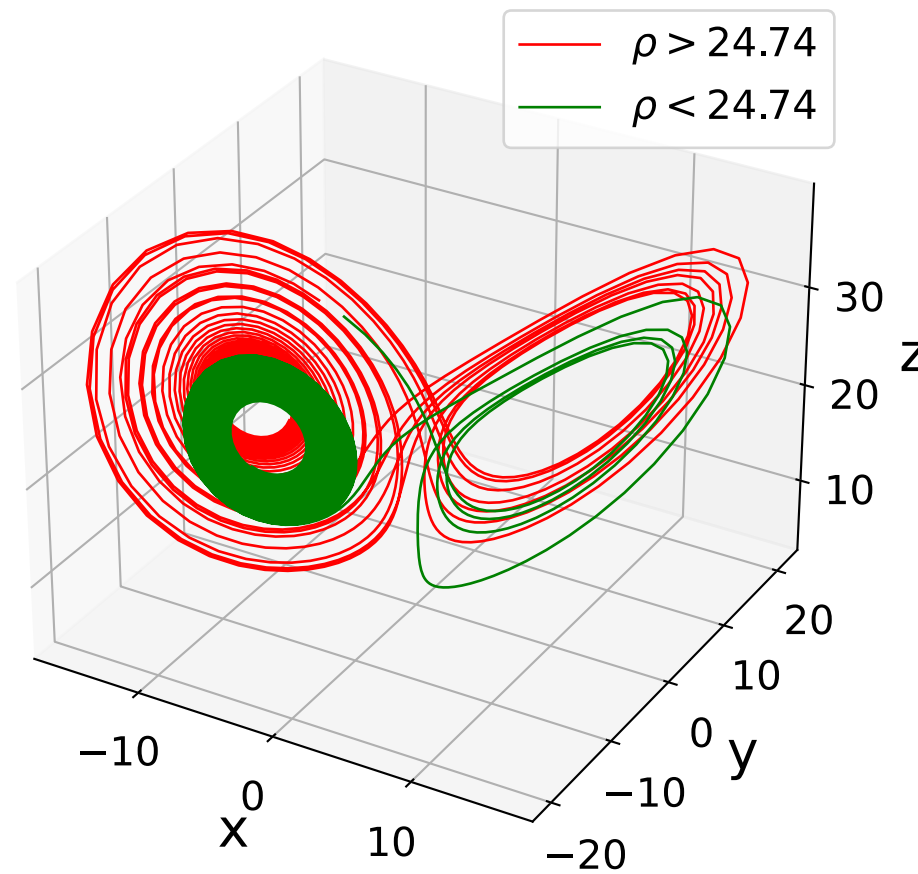

Supplement: Supplementary 1 — Appendix A to G Figs. S1 to S10 Tables S1 to S5 [file research.0174.f1.zip › Fig-S4.pdf]

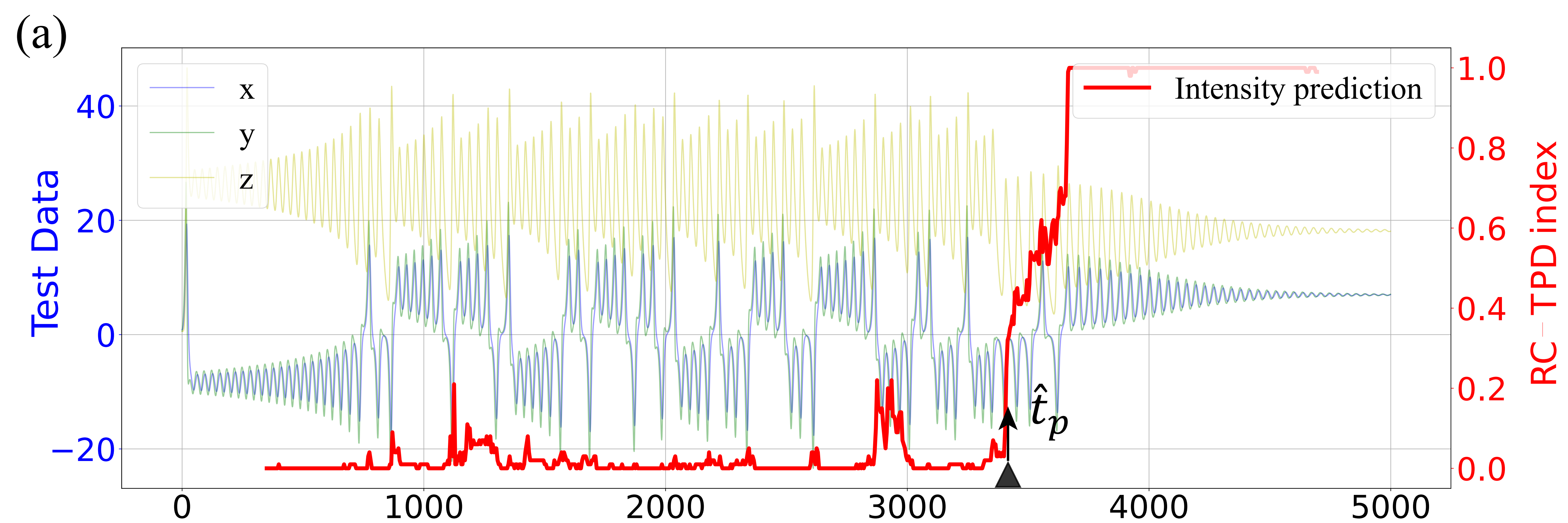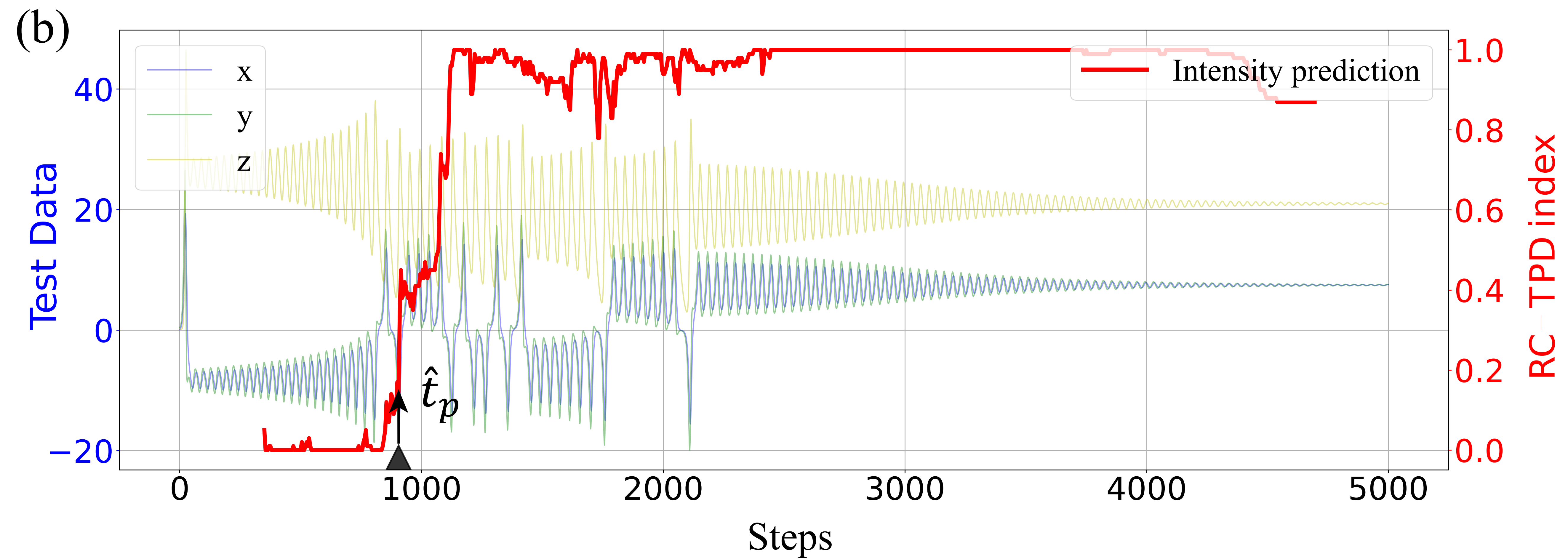

Supplement: Supplementary 1 — Appendix A to G Figs. S1 to S10 Tables S1 to S5 [file research.0174.f1.zip › Fig-S5.pdf]

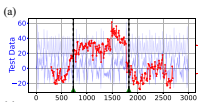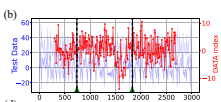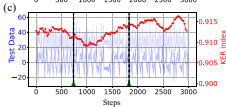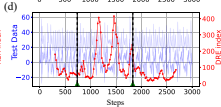

Supplement: Supplementary 1 — Appendix A to G Figs. S1 to S10 Tables S1 to S5 [file research.0174.f1.zip › Fig-S6.pdf]

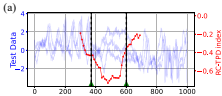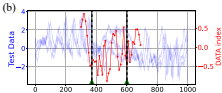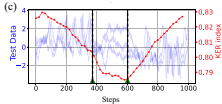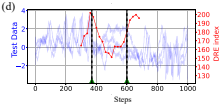

Supplement: Supplementary 1 — Appendix A to G Figs. S1 to S10 Tables S1 to S5 [file research.0174.f1.zip › Fig-S7.pdf]

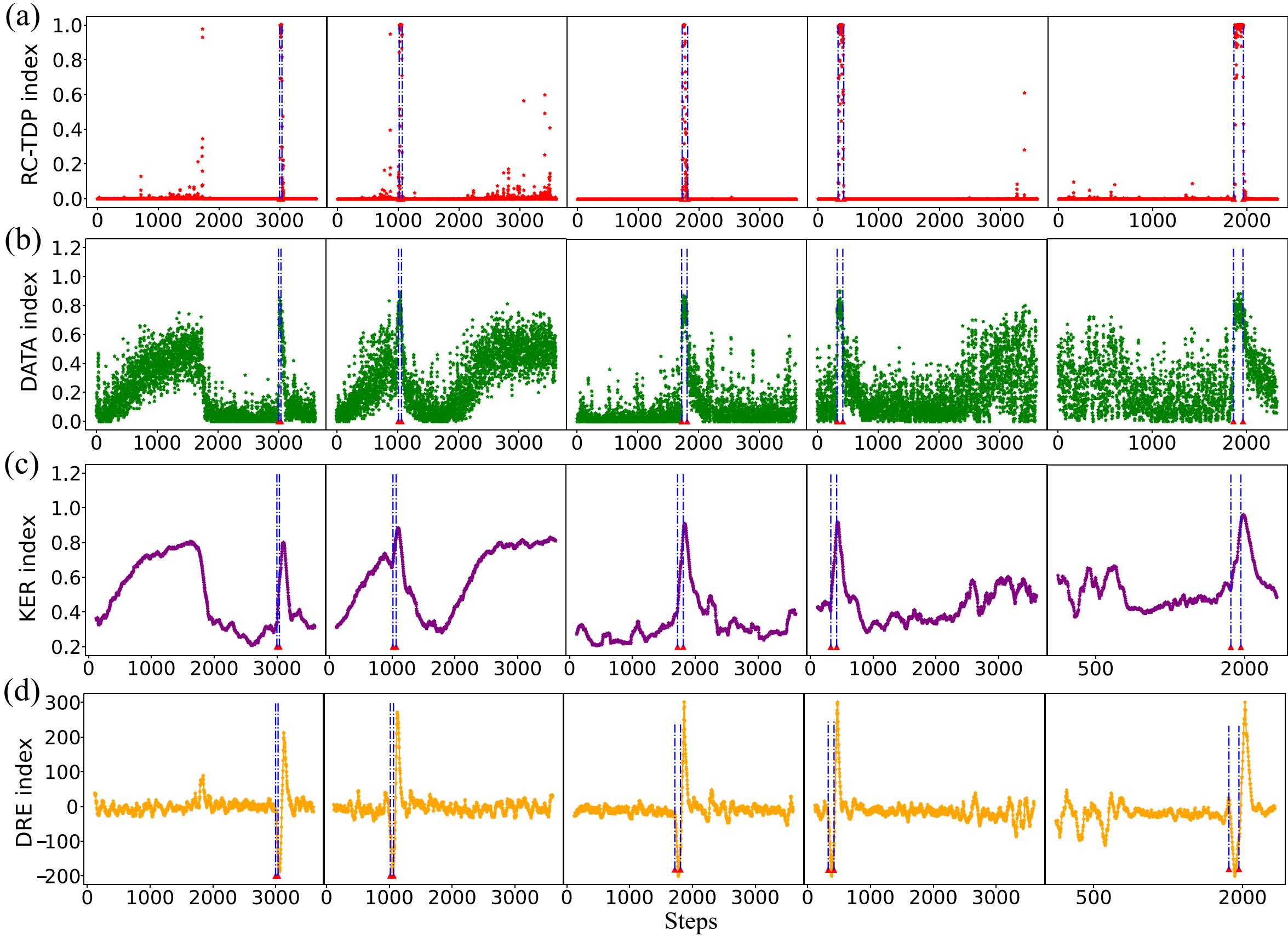

Supplement: Supplementary 1 — Appendix A to G Figs. S1 to S10 Tables S1 to S5 [file research.0174.f1.zip › Fig-S8.pdf]

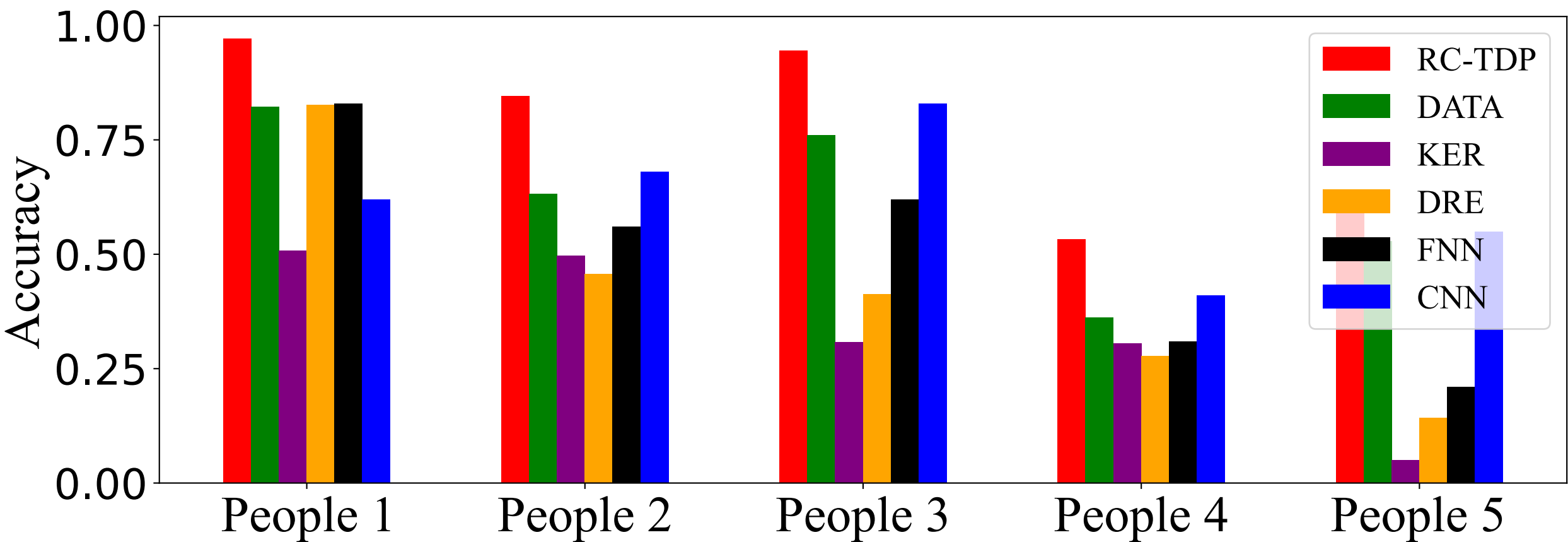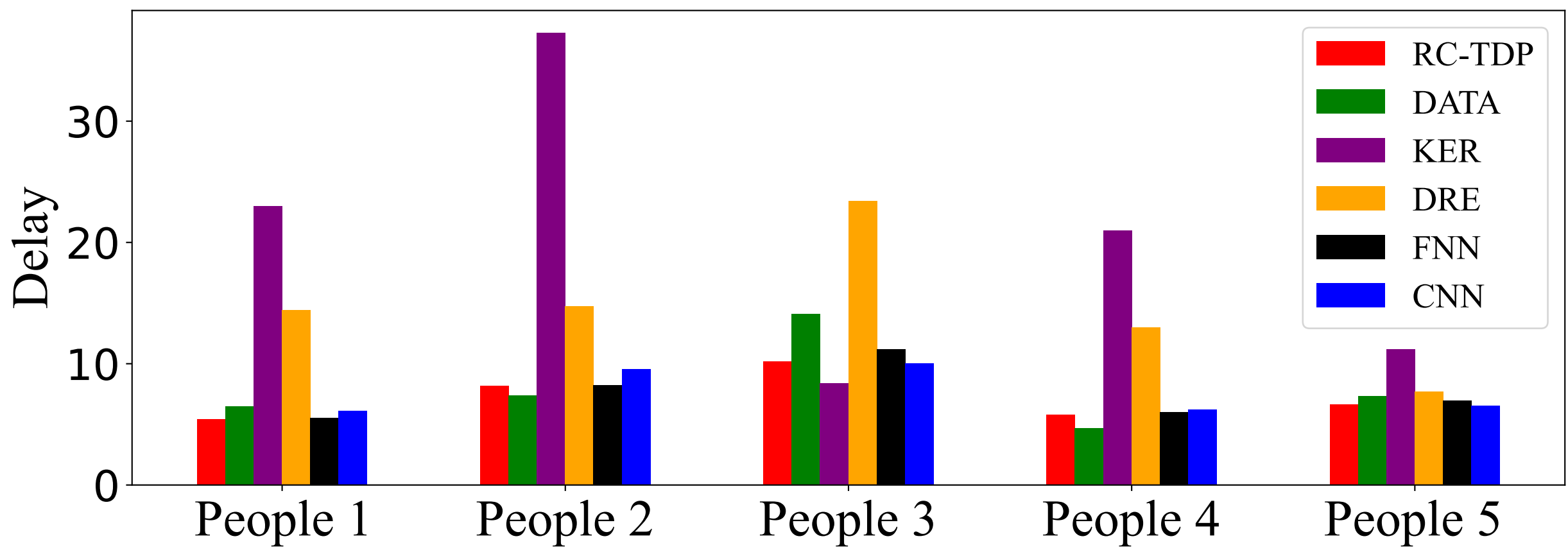

Supplement: Supplementary 1 — Appendix A to G Figs. S1 to S10 Tables S1 to S5 [file research.0174.f1.zip › Fig-S9.pdf]

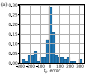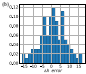

Supplement: Supplementary 1 — Appendix A to G Figs. S1 to S10 Tables S1 to S5 [file research.0174.f1.zip › Fig-S10.pdf]
